# Should Virtual Objective Structured Clinical Examination (OSCE) Teaching Replace or Complement Face-to-Face Teaching in the Post-COVID-19 Educational Environment: An Evaluation of an Innovative National COVID-19 Teaching Programme

**DOI:** 10.7759/cureus.49708

**Published:** 2023-11-30

**Authors:** Charles Gamble, Alice Oatham, Raj Parikh

**Affiliations:** 1 Trauma and Orthopaedics, Royal Stoke Hospital, Stoke-on-Trent, GBR; 2 Obstetrics and Gynaecology, Chesterfield Royal Hospital, Chesterfield, GBR; 3 Geriatrics, Royal Oldham Hospital, Northern Care Alliance, Manchester, GBR

**Keywords:** medical education, final year examinations, medical student teaching, covid-19, near-peer teaching, virtual medical teaching, objective structured clinical exam (osce)

## Abstract

Background

The COVID-19 pandemic brought about drastic changes to medical education and examinations, with a shift to online lectures and webinars. Additionally, social restrictions in the United Kingdom (UK) inhibited students’ ability to practice for objective structured clinical examination (OSCE) with their peers.

Methods

The Virtual OSCE buddy scheme (VOBS) provided a means to practice OSCE skills virtually by linking groups of 2-6 final-year medical students with a junior doctor who had recently passed their exams. Sessions were held virtually, tailored to the needs of each group, in a 3-month period prior to examinations. The scheme ran across two examination periods, 2020/21 and 2021/22, including a total of 13 universities throughout the UK.

Results

In 2020/21, 96% (n=227) of students described improved confidence in OSCE scenarios. Furthermore, 90% (n=213) reported improvement in communication, 89% (n=211) in diagnosis and clinical reasoning and 86% (n=203) in history-taking skills. Examination and procedural skills proved more challenging to practice virtually, with improvement reported by 31% (n=73) and 15% (n=36) of students, respectively. Ninety-three per cent (n=58) of buddies reported improved lesson planning abilities and 90% (n=57) felt more confident in their teaching.

In 2021/22, 90% (n=133) of students felt more prepared for their OSCE. In key skills, improvement was reported by 87% (n=128) in communication, 84% (n=124) in diagnosis and clinical reasoning and 83% (n=123) in history-taking. In this cohort, 40% (n=59) reported improvement in examination skills and 24% (n=36) in procedural skills. Ninety per cent (n=83) of buddies reported an improvement in teaching skills, with 93% (n=85) increasing their confidence to teach.

Conclusion

VOBS demonstrates the benefits to students and teachers of near-peer OSCE teaching. Given the virtual nature, the main drawback is the inability to practice hands-on examination and procedural skills. This scheme provides insight to educators planning virtual teaching programmes in the future. With the evolution of technology, virtual examination and procedure practice may be possible in the near future. VOBS would suggest that currently, virtual OSCE teaching should be used to complement face-to-face teaching.

## Introduction

When the United Kingdom (UK) was placed into lockdown in March 2020 this brought about sudden and major changes to medical education and examinations. Medical schools had to rapidly adapt their teaching methods, resulting in increased use of online resources [1]. Online resources allow for increased flexibility, however, students reported the effectiveness of clinical teaching online was reduced compared to face-to-face contact [2]. COVID-19 restrictions had significant effects on those nearing the end of medical school and about to begin as Foundation Year 1 (FY1) doctors. In a national survey, 59.3% and 22.7% of students felt less prepared and less confident to start FY1, respectively [3]. Objective structured clinical examinations (OSCEs) are an essential element of medical education and are used throughout UK medical schools to assess performance and identify those who may struggle in the clinical environment [4]. Given the nature of the examination, it is common for medical students to practice OSCEs in person. Restrictions applied during the COVID-19 pandemic meant that face-to-face practice was significantly reduced.

Online OSCE practice is a relatively novel phenomenon. It is common for medical students to practice together or to rely on mock OSCEs for their preparation. Robinson et al. found that mock OSCEs improved medical students’ confidence and significantly reduced anxiety related to the examinations [5]. In a small study at Southampton University peer-led mock OSCEs were reported as ‘useful’ in preparation for examinations, and students reported increased confidence and expected performance and it helped guide further revision [6]. However, online alternatives have become increasingly utilised since COVID-19 with Motkur et al. finding online practice to be more accessible, efficient and convenient, yet lacked benefits for clinical assessment stations. Overall, Motkur et al. concluded that students were not willing to replace face-to-face teaching with online alternatives, however, some students reported that the two could be used together [7]. Hannan et al. designed and organised an OSCE using ‘Zoom’ and reported positive results, with one participant describing the experience as ‘very efficient and smooth’ [8].

This project is an example of near-peer teaching. Near peer, a term coined by Whitmann in 1988, describes peers of 1-2 years seniority teaching junior colleagues [9]. It is beneficial as the material is pitched at the correct level as the tutors appreciate the learners’ needs and perspectives. Students value the insight and contributions of doctors who have recently tackled the same examination hurdles [10].

This paper presents data from a national virtual OSCE teaching scheme that ran across the 2020/21 and 2021/22 examination periods and involved students from 13 Universities. It was an online programme that facilitated near-peer teaching. We aim to highlight the positives and negatives of this style of teaching for a practical examination and provide insights for medical educators in an ever-evolving online educational environment.

## Materials and methods

This descriptive paper presents results from participant feedback survey from a national teaching programme called the virtual OSCE buddy scheme (VOBS). VOBS was designed by graduates of UK medical schools in 2020 in response to COVID-19 restrictions limiting face-to-face practice for final-year medical students. The scheme offered final-year students a means to practice their OSCE skills virtually by linking groups of 2-6 final-year medical students with a ‘buddy’. Buddies were junior doctors who had recently passed their final-year examinations and were currently working in the National Health Service (NHS). Buddies and medical students were recruited through email circulation, private social media groups and word of mouth.

Over a period of 3 to 5 months, buddies were encouraged to hold weekly sessions. They worked with their group to consider key OSCE stations, provide feedback and promote reflection. Teaching was delivered via online videocall platforms. The topics of each session were guided by the specific needs of the group. A selection of key OSCE stations were available if required.

The scheme commenced in late 2020 and started in Nottingham. Initially, 268 students and 93 buddies were recruited. Sessions were delivered over a period of 5 months from November 2020 to March 2021. The initial success of the Nottingham scheme meant that VOBS was expanded to four other medical school catchment regions (The University of Manchester, The University of Oxford, Imperial College London and University College London).

In the first year, the VOBS scheme was responsible for conducting sessions with 680 students via 242 buddies. Following this success, the scheme was expanded in its second year (the 2021/22 academic year) to include a total of 13 Universities. At each university, buddies were encouraged to hold a minimum of six sessions across a 12-week period in the build-up to their students’ final-year OSCE. There were a small number of buddies who participated in both years of the scheme, however, the set of students was entirely different across the two years.

Data was collected via post-scheme feedback surveys, one for students and one for buddies. Google Forms was used to deliver the feedback forms and collate responses. The forms utilised yes/no questions as well as Likert scales to help explore the outcomes for both students and buddies. No pilot survey was used. Feedback forms were sent to participants near each university’s final-year OSCE date, with regular reminders being sent to encourage completion.

There was a slight variation in the feedback forms across the two years. To make feedback more user-friendly the Likert scale wording used in the second year of the scheme was adapted. In 2020/21 ratings initially read 1 (not a lot) to 5 (a lot) considering whether the scheme had improved learners’ medical knowledge, confidence in OSCE skills and improvement in key outcomes (history-taking, examination skills, diagnosis and clinical reasoning, communication, and procedural skills). In 2021/22, the wording changed to one based on agreement with statements with 1 (strongly disagree) to 5 (strongly agree). At the end, learners were asked to rate the utility of the scheme using a Likert scale.

Buddies were asked questions in a similar format. Questions considered whether VOBS had improved teaching skills, communication, lesson planning and confidence to lead sessions. The wording was changed in the second year of the scheme mirroring the change for learners. Buddies were asked if they enjoyed participating and if the scheme was rewarding.

## Results

**Figure 1:**
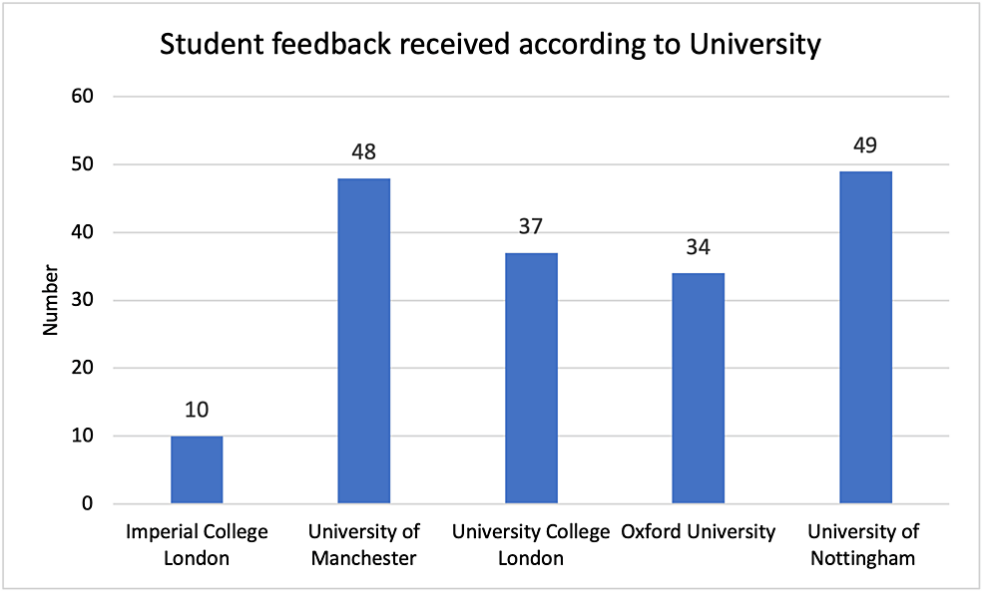
Student feedback 2020/21, number per university.

**Figure 2:**
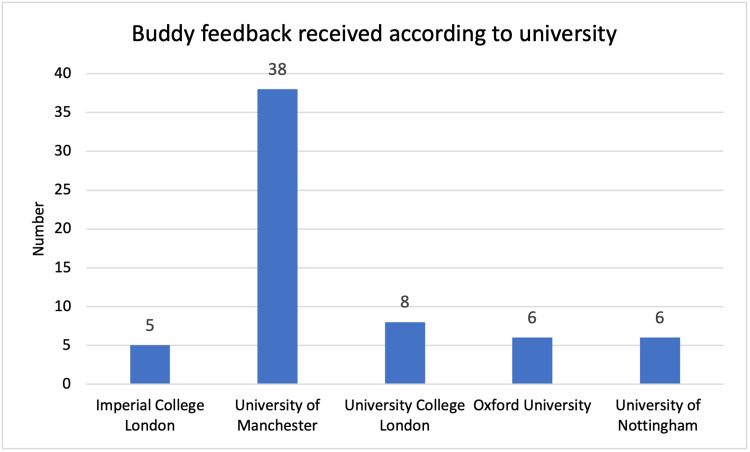
Buddy feedback 2020/21, number per university.

**Figure 3:**
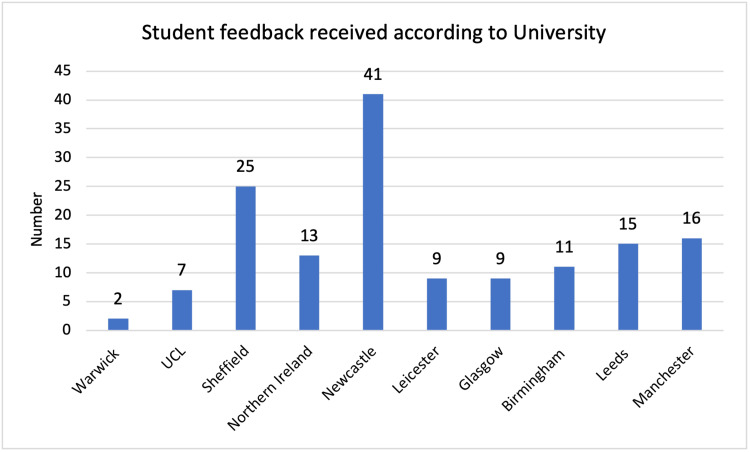
Student feedback 2021/22, number per university.

**Figure 4:**
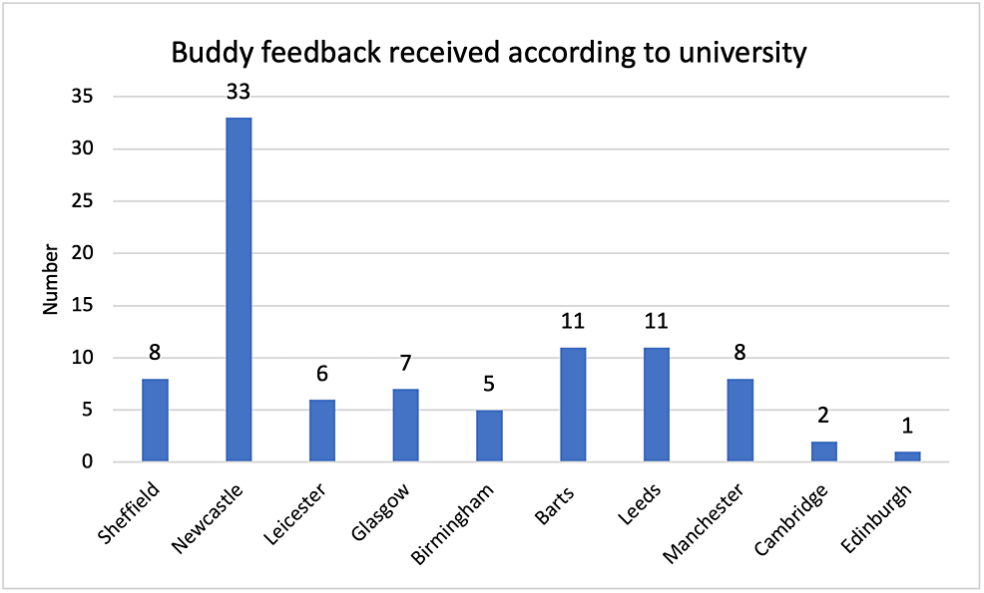
Buddy feedback 2021/22, number per university.

The total feedback numbers received from each university can be seen for students and buddies, across the two years in Figures 1-4. In the 2020/21 scheme, five universities were involved. In the second year, a further eight universities were involved. Feedback numbers were variable between universities. Collecting feedback once the scheme was completed proved a significant challenge to continue engagement once students had finished their OSCE. It can also be seen that at some universities’ feedback was only received from students or buddies.

2020-2021 students

A total of 236 of 680 (34.7%) students participated in post-scheme feedback. Of those providing feedback, 86% felt that VOBS positively improved (score 4 or 5) their medical knowledge and 96% felt that VOBS had increased their confidence (score 4 or 5) in OSCE skills. In the key outcomes scores of 4 or 5 (suggesting improvement) were reported by 86% in history-taking skills, 31% in examination skills, 89% in diagnosis and clinical reasoning, 90% in communication skills and 15% in procedural skills.

**Figure 5:**
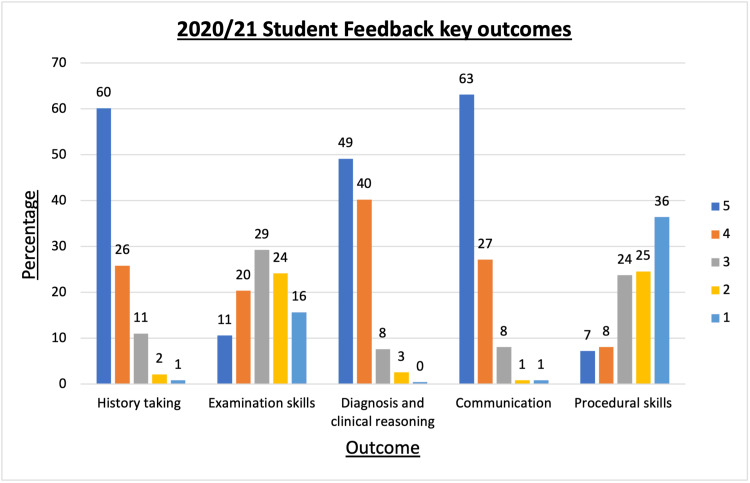
Percentage scores on Likert scale 1 (not a lot) to 5 (a lot) for student feedback when asked how much VOBS had improved key OSCE skills. OSCE: objective structured clinical examination

The spread of responses is illustrated in Figure 5. Overall, 98% of students reported that they found the scheme useful.

2020-2021 buddies

A total of 63 out of 242 (26%) buddies filled out post-scheme feedback. In the key outcomes, scores of 4 or 5 (signifying improvement) were given by 87% in teaching skills, 78% in communication skills, 92% in lesson planning and 91% in teaching confidence.

**Figure 6:**
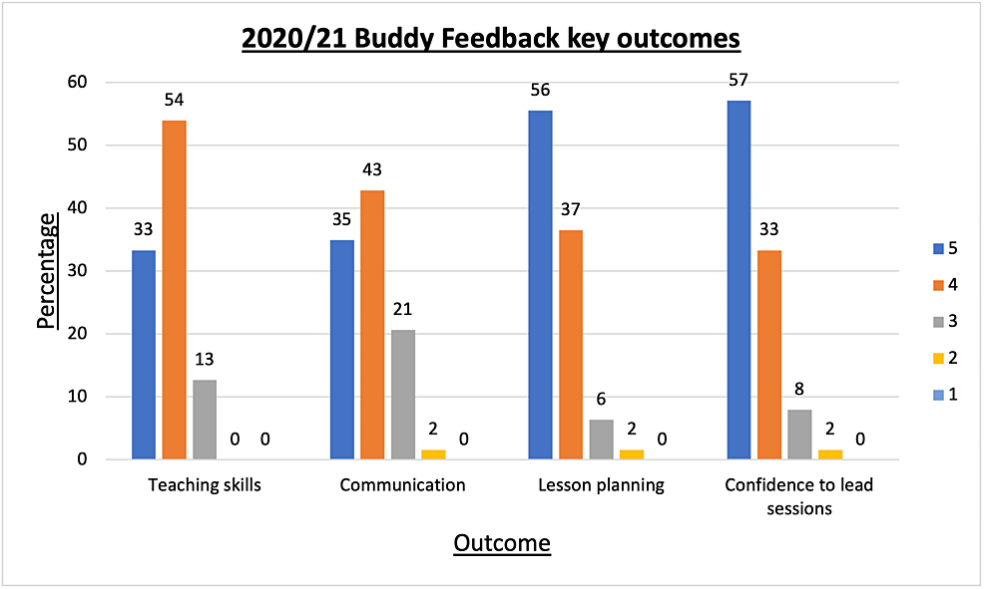
Percentage scores on Likert scale 1 (not a lot) to 5 (a lot) for buddy feedback when asked how much VOBS had improved key teaching skills. VOBS: Virtual objective structured clinical examination buddy scheme

The spread of responses is illustrated in Figure 6. All participants reported that they enjoyed the scheme and found it rewarding.

2021-2022 students

A total of 148 out of 417 (35%) students participated in post-scheme feedback. Eighty-seven percent strongly agreed or agreed that VOBS improved their medical knowledge and 90% felt that VOBS had helped them prepare for the final-year OSCE.

**Figure 7:**
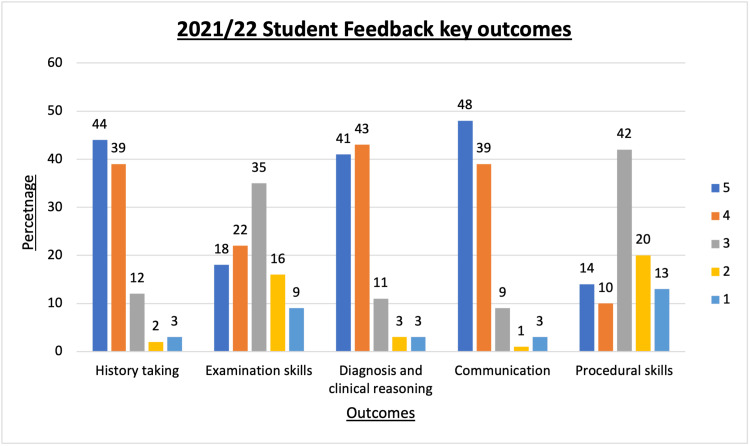
Percentage scores on Likert scale 1 (strongly disagree) to 5 (strongly agree) for student feedback when asked if VOBS had improved key OSCE skills. VOBS: Virtual OSCE buddy scheme; OSCE: objective structured clinical examination

In the key outcomes, the percentage of learners who strongly agreed or agreed that they had improved regarding specific outcomes were as follows: 83% in history-taking skills, 40% in examination skills, 84% in diagnosis and clinical reasoning, 87 % in communication skills and 24% in procedural skills. The spread of responses is illustrated in Figure 7. Overall, 94% of students reported that they found the scheme useful.

2021-2022 buddies

A total of 92 out of 185 (50%) buddies filled out post-scheme feedback. In the key outcomes, 90% strongly agreed or agreed they had improved their teaching skills, 82% reported improved communication skills, 88% reported improvement in lesson planning and 93% reported improvement in teaching confidence.

**Figure 8:**
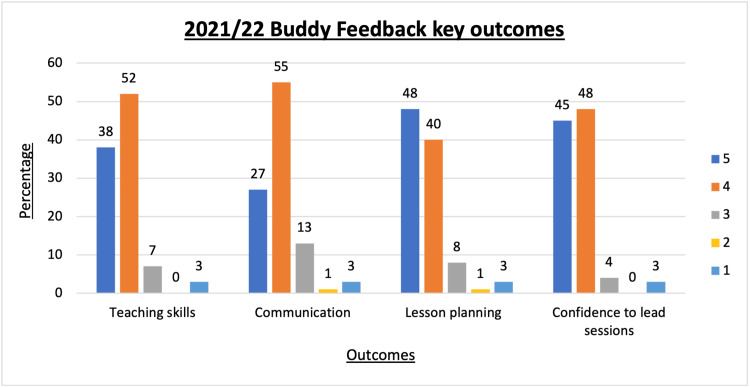
Percentage scores on Likert scale 1 (strongly disagree) to 5 (strongly agree) for buddy feedback when asked if VOBS had improved key teaching skills. VOBS: Virtual objective structured clinical examination buddy scheme

The spread of responses is illustrated in Figure 8. All participants found the scheme rewarding to participate in and all but one reported that they enjoyed participating.

## Discussion

This scheme has highlighted that a virtual near-peer OSCE practice programme can have benefits for students and teachers. Across both years of the scheme, over 80% of students reported improvement in their history-taking, diagnosis and clinical reasoning and communication skills. Over 86% felt their medical knowledge had improved and in the second year of the scheme, 90% of students felt better prepared for their OSCE following completion of the teaching programme. Our findings are consistent with examples in the current literature of near-peer schemes. The majority of 114 medical students reported improved knowledge following a near-peer programme covering respiratory pathology [11]. Similarly, in a UK-based near-peer revision series covering eight common clinical presentations, 93% of students reported increased confidence in recognition and management of each condition [12].

Teachers, or buddies, also reported significant benefits. Across the two years of the scheme over 78% of buddies reported improvement in all key outcome measures; teaching skills, communication, lesson planning and confidence to run teaching sessions. Furthermore, buddies were encouraged to tailor their sessions to the needs of students, meaning they would need to adapt their teaching plans and style for the individual needs of their group. A systematic review by Tanveer et al. of articles between 2012-2022 reported improvement in knowledge retention, teaching skills, leadership, communication and confidence for teachers in near-peer teaching programmes [13]. Furthermore, teachers in a peer-assisted learning programme were seen to perform better in anatomy examinations when compared to the learners of the same programme, demonstrating the clear benefit for the teachers as well as the learners [14].

There were, however, some drawbacks for the students. Examination skills were reported as improved by only 31% and 40% and procedural skills by 15% and 24% in 2020/21 and 2021/22, respectively. This finding is unsurprising given that the teaching was delivered via online platforms, making examinations and procedural skills very difficult to practice. Simulation has been widely used in medical education and could provide a realistic option to remedy this. Simulation training has been shown to benefit surgical trainees in laparoscopic skills and Schroedl et al. demonstrated that simulator-trained residents performed significantly higher in bedside assessments than those trained traditionally in the medical intensive care unit [15-16]. With regard to medical student education, Zhao et al. performed a meta-analysis of the use of virtual reality in anatomy teaching and reported that virtual reality improved learners’ knowledge of anatomy [17]. This highlights a potential area for investigation in future studies, with continued advancement in technology there may be a greater ability to provide realistic examination and procedural skill practice virtually for OSCE preparation.

The findings of this project are similarly aligned with existing literature regarding near-peer OSCE teaching. In a study evaluating near-peer teaching for penultimate-year medical students at The University of Manchester, key OSCE topics were identified from the syllabus and a series of lectures were provided covering these topics [18]. Results showed a significant increase in mean confidence levels and students found the programme valuable in guiding their own revision. The benefit of near-peer teaching for OSCE revision has previously been highlighted by Rashid et al [19]. All students in this study found near-peer OSCE teaching to improve their confidence leading up to exams. Additionally, 73.2% agreed or strongly agreed that the near-peer teaching was comparable to that of consultants.

This study does have some limitations. First, the feedback received was of perceived improvement in the key outcome areas. There was no objective assessment to evaluate the improvement or a positive impact on examination results. Future projects should attempt to incorporate this. Second, given that the project ran across 13 universities, the teaching given was not able to be standardised. Each buddy was encouraged to guide their sessions based on the individual needs of the students in their group, but this does allow for potential variability in the quality of teaching each student group received. The sessions were small group sessions, run at the convenience of each group, meaning that sessions were not observed by one individual to compare or confirm participation.

Additionally, data was not collected to highlight any participants in the scheme who withdrew during the teaching programme and thus the total number of participants as stated may not be equivalent to those that were actively participating at the time of data collection. Last, a variety of popular online meeting platforms were utilised scheme-wide, at the discretion of buddies following suggestions from committee members. No standardised teaching platform was consistently used, nor endorsed, which could pose issues for reproducibility.

## Conclusions

The VOBS demonstrates clear benefits to both students and teachers of virtual near-peer OSCE practice. Students noted improvement in key OSCE skills, gaining confidence and medical knowledge during the scheme. Buddies felt their teaching abilities improved, including lesson planning and confidence in delivery. As discussed, the key limitation of a virtual scheme is the challenges posed by tactile feedback and live practice of OSCE scenarios.

This study suggests that virtual OSCE teaching is beneficial but is unlikely to be able to replace face-to-face teaching, therefore suggesting that virtual teaching should complement face-to-face OSCE teaching.
